# Characterization of Metabolic Changes under Low Mineral Supply (N, K, or Mg) and Supplemental LED Lighting (Red, Blue, or Red–Blue Combination) in *Perilla frutescens* Using a Metabolomics Approach

**DOI:** 10.3390/molecules25204714

**Published:** 2020-10-14

**Authors:** Dong Ho Suh, Yangmin X. Kim, Eun Sung Jung, Seulbi Lee, Jinyong Park, Choong Hwan Lee, Jwakyung Sung

**Affiliations:** 1 Department of Bioscience and Biotechnology, Konkuk University, Seoul 05029, Korea; sdh14031988@naver.com (D.H.S.); park4jy@hanmail.net (J.P.); 2 National Institute of Agricultural Sciences, Rural Development Administration, Wanju 55365, Korea; yangmink@korea.kr (Y.X.K.); seulvi23@korea.kr (S.L.); 3 Department of Systems Biotechnology, Konkuk University, Seoul 05029, Korea; jes708@naver.com; 4 Research Institute for Bioactive-Metabolome Network, Konkuk University, Seoul 05029, Korea; 5 Department of Crop Science, College of Agriculture, Life and Environment Sciences, Chungbuk National University, Cheongju 28644, Korea

**Keywords:** LED lighting, metabolite profiling, mineral nutrient, perilla

## Abstract

In order to achieve premium quality with crop production, techniques involving the adjustment of nutrient supply and/or supplemental lighting with specific light quality have been applied. To examine the effects of low mineral supply and supplemental lighting, we performed non-targeted metabolite profiling of leaves and stems of the medicinal herb *Perilla frutescens*, grown under a lower (0.75×) and lowest (0.1×) supply of different minerals (N, K, or Mg) and under supplemental light-emitting diode (LED) lighting (red, blue, or red–blue combination). The lowest N supply increased flavonoids, and the lowest K or Mg slightly increased rosmarinic acid and some flavonoids in the leaves and stems. Supplemental LED lighting conditions (red, blue, or red–blue combination) significantly increased the contents of chlorophyll, most cinnamic acid derivatives, and rosmarinic acid in the leaves. LED lighting with either blue or the red–blue combination increased antioxidant activity compared with the control group without LED supplementation. The present study demonstrates that the cultivation of *P. frutescens* under low mineral supply and supplemental LED lighting conditions affected metabolic compositions, and we carefully suggest that an adjustment of minerals and light sources could be applied to enhance the levels of targeted metabolites in perilla.

## 1. Introduction

*Perilla frutescens*, belonging to the family Lamiaceae, is a native plant used as a dietary and medicinal herb in Southeast Asian countries such as Korea, China, and Japan, as well as other regions of Asia. The leaves of *P. frutescens* have been used as a traditional herbal medicine to treat numerous disorders, such as asthma, cough, colds, and allergies [1]. Recently, many researchers carried out substantial investigations into the phytochemistry of *P. frutescens*. Several studies reported that many bioactive metabolites were found in *P. frutescens*, including flavonoids, fatty acids, triterpenes, and phenolic compounds [1,2], which have potent antioxidant and antiallergic activities as well as growth inhibitory activity against cancer cells [3,4,5].

In recent times, various edible or medicinal plants were cultivated in greenhouses under controlled environmental conditions because this is a way to not only increase the yield but also improve the nutritional value. To control the quality of cultivated plants in a greenhouse, various environmental factors can be adjusted to regulate plant metabolism, while these factors can cause stress to plants [6]. A low supply of mineral fertilizer was used as a strategy to improve crop quality [7]. Nitrogen (N), potassium (K), and magnesium (Mg) are among the most important minerals required by plants for nutrition, and understanding the effects of low mineral nutrient supply on plant metabolism is important for agriculture [8]. Because of their various roles in plant development and growth, low N, K, or Mg supply can directly or indirectly affect plant metabolic pathways. Light is an environmental factor that is essential for the growth and development of plants. There has been a wealth of research on the effects of light-emitting diode (LED) lighting on plant growth and development for the purposes of enhancing harvest productivity and plant quality [9]. Therefore, it is important to understand how low mineral supply and supplemental LED lighting conditions influence the synthesis of many bioactive compounds in plants as well as plant metabolism. Primary and secondary metabolites are the intermediate or final products of intricate networks of biochemical pathways involved in plant metabolism. However, an overview of the overall metabolic pathway underlying plant responses to different mineral deficiencies and supplemental LED lighting conditions is still lacking. By using a metabolomics approach, we can enable a more detailed understanding of which metabolites change in response to mineral deficiency and supplemental LED lighting conditions, and how these changes are similar or differ among various cultivation conditions, which will increase our understanding of plant nutrient responses, interactions between metabolic networks, and basic plant metabolism.

Plant metabolomics can be used to generate a holistic picture of the overall metabolic changes in plants according to various environmental stresses. Environmental stresses (drought, high salinity, temperature, and mineral nutrient deficiency) in plants can change the growth conditions, causing an adjustment of the metabolic pathway referred to as acclimation [10]. Plant metabolomics were used to study various stress conditions in plants, such as temperature, salinity, oxidative stress, and multiple stresses [11,12,13,14]. The main objective of the present study is to understand plant metabolism under different conditions of mineral deficiency and supplemental LED in the leaves and stems of *P. frutescens*.

## 2. Results

### 2.1. Physicochemical Parameters and Metabolic Variations under Different Mineral Supply Conditions in Leaves and Stems of P. frutescens

**Figure 1:**
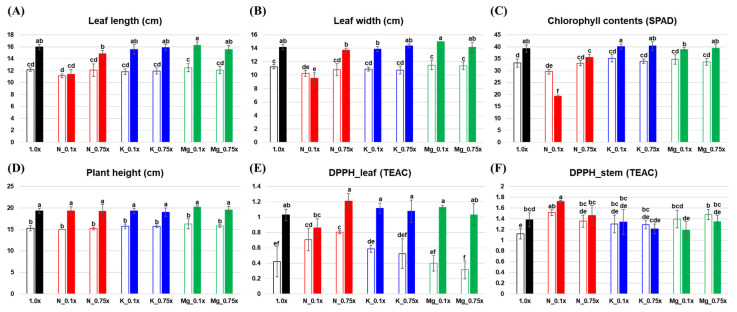
Leaf length (**A**), leaf width (**B**), chlorophyll content (**C**), plant height (**D**), and antioxidant activity (**E**,**F**) under different mineral conditions in *Perilla frutescens*. Empty bars represent 10 days of low mineral supply, and filled bars represent 21 days of low mineral supply. Different letters indicate significant differences according to Duncan’s multiple-range test by analysis of variance (ANOVA, *p*-value < 0.05). TEAC: trolox equivalent antioxidant capacity; N: nitrogen; K: potassium; Mg: magnesium.

Various physicochemical parameters, namely leaf length, leaf width, chlorophyll content, plant height, and antioxidant activity, were measured under different mineral supply conditions in *P. frutescens* (Figure 1). In the control group, the chlorophyll content, plant height, and antioxidant activity significantly increased from 10 to 21 days of experimental time period, in which plants grew and their antioxidant activity increased. After 10 days with different levels of nutrient supply, there was no significant difference in leaf length, leaf width, chlorophyll content, or plant height. However, 2,2-diphenyl-1-picrylhydrazyl (DPPH) levels in the leaves were significantly higher under low N supply (N_0.1×, N_0.75×) than in the control, and DPPH in the stem was higher in N_0.1×, Mg_0.1×, and Mg_0.75× than in the control. After a prolonged period with different levels of nutrient supply (21 days), N_0.1× showed significantly reduced leaf length and leaf width, and low N supply (N_0.1×, N_0.75×) showed significantly reduced chlorophyll content; DPPH in the leaves was not dependent on the nutrient supply, but DPPH in the stem was significantly higher in N_0.1× than in the control.

**Figure 2:**
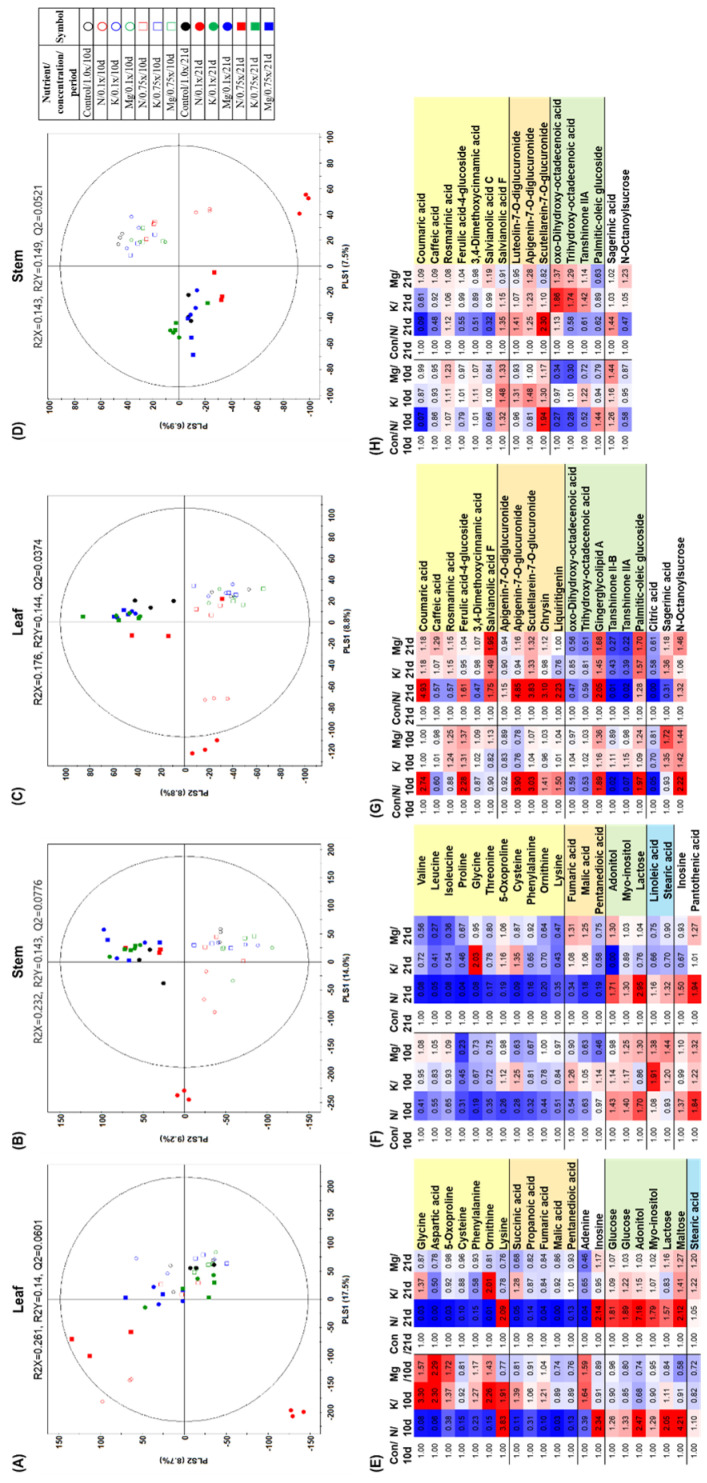
Partial least squares-discriminate analysis (PLS-DA) score plots derived from GC–TOF-MS datasets (**A**,**B**) and UHPLC–LTQ-MS/MS datasets (**C**,**D**) under different mineral supply conditions in *Perilla frutescens*. For the lowest mineral supply condition (0.1×), significantly altered metabolites (VIP > 1.0 and *p*-value < 0.05) are represented by fold change from GC–TOF-MS datasets for leaves (**E**) and stems (**F**) and UHPLC–LTQ-MS/MS datasets for leaves (**G**) and stems (**H**). Con: control; N: nitrogen; K: potassium; Mg: magnesium.

To investigate the effects of different mineral supply conditions on metabolites in the leaves and stems of *P. frutescens*, we performed non-targeted metabolite profiling using gas chromatography–time of flight mass spectrometry (GC–TOF-MS) and ultra-high-performance liquid chromatography–linear trap quadrupole tandem mass spectrometry (UHPLC–LTQ-MS/MS) with multivariate statistical analysis. The partial least squares-discriminate analysis (PLS-DA) score plot from the GC–TOF-MS dataset for leaves (Figure 2A) exhibited an overlap between 10- and 21-day experiment samples, while other PLS-DA score plots from the GC–TOF-MS dataset for stems and the UHPLC–LTQ-MS/MS dataset for leaves and stems (Figure 2B–D) were clearly distinct for the two timepoints (10 and 21 days) and clustered according to each experimental group. Among them, the N_0.1× condition was clearly separated from the other supply conditions in the PLS-DA score plots.

To further explain the effects of mineral supply, the metabolites that were significantly altered under the lowest mineral supply condition (0.1×) were identified based on having a variable importance in projection (VIP) value of >1.0 and *p* < 0.05. According to these criteria, a total of 58 metabolites were identified as significantly altered, including 12 amino acids, 6 carbohydrates, 5 organic acids, 2 purines and pyrimidines, 7 cinnamic acid derivatives, 6 flavonoids, 6 lipids, 4 unsorted metabolites, 1 fatty acid, and 9 unidentified metabolites (Tables S1 and S2). The N_0.1× condition derived from the GC–TOF-MS dataset (Figure 2E,F) showed that the relative abundance of most amino acids and organic acids, except lysine in leaves, decreased in both leaves and stems, while the levels of some carbohydrates increased in comparison with the control. The levels of lipids and their derivatives decreased under all mineral conditions in leaves and stems (Figure 2G,H); however, some flavonoids in leaves accumulated only under the N_0.1× condition.

### 2.2. Comparison of Results for Metabolic Pathway Analysis under Different Mineral Supply Conditions

**Figure 3:**
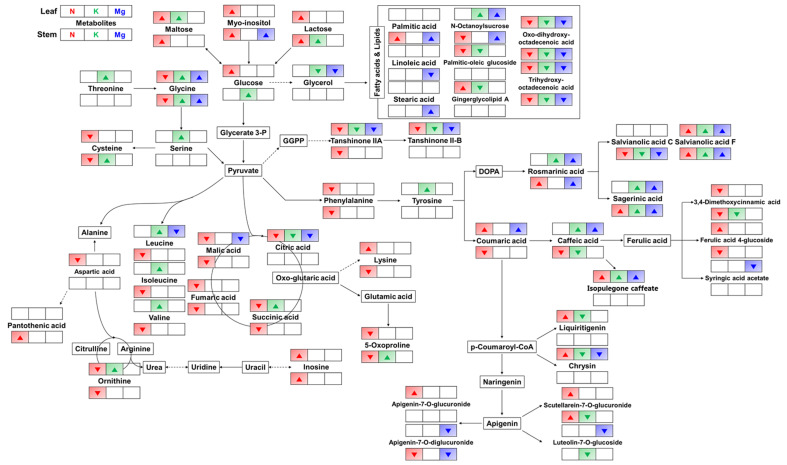
The proposed metabolic pathway under the lowest mineral supply for 21 days in *Perilla frutescens*. Significantly altered metabolites are represented by up and down arrows for each mineral-deficient condition depending on whether their levels increased or decreased, respectively. The pathway was modified from KEGG (http://www.genome.jp/kegg). N: nitrogen; K: potassium; Mg: magnesium.

To further understand the metabolic perturbation caused by the lowest mineral supply condition (0.1×), we also conducted metabolic pathway analysis in both leaves and stems based on the list of identified metabolites (Figure 3). According to metabolic pathway, the relative levels of most metabolites showed similar changes for both leaves and stems. Most carbohydrates (maltose, myo-inositol, lactose, and glucose) corresponding to the initial steps of glycolysis increased under the N_0.1× condition compared with the control group. For the metabolism of amino acids derived from pyruvate, the levels of most amino acids significantly decreased under the N_0.1× condition, while some amino acids, including serine, glycine, threonine, leucine, isoleucine, valine, tyrosine, and ornithine, had accumulated in leaves under the K_0.1× condition. Consequentially, most organic acids related to the tricarboxylic acid (TCA) cycle, including malic acid, fumaric acid, succinic acid, and citric acid, were downregulated under the N_0.1× condition, while some organic acids (malic acid and citric acid) were decreased under the Mg_0.1× condition compared with the control group. According to the metabolic pathway of the metabolism of fatty acids and lipids derived from glycerol, the levels of oxo-dihydroxy-octadecenoic acid and trihydroxy-octadecenoic acid decreased under all mineral-deficient conditions compared with the control, and other fatty acids showed disparate patterns.

In the case of secondary metabolism, most of the flavonoids and cinnamic acid derivatives that were present in the examined *P. frutescens* were synthesized through the shikimate pathway. The levels of phenylalanine and tyrosine, which are the main precursors of the shikimate pathway, were downregulated under the N_0.1× condition and increased only in leaves under the K_0.1× condition. After that, most of the low mineral supply conditions showed the accumulation of rosmarinic acid derivatives (rosmarinic acid, sagerinic acid, and salvianolic acid F) derived from 3,4-dihydroxyphenylalanine (DOPA), while the level of salvianolic acid C was downregulated only in stems under all low mineral supply conditions. Most caffeic acid derivatives, which are biosynthesized from coumaric acid, showed different patterns between leaves and stems under different mineral supply conditions. Among them, caffeic acid, 3,4-dimethoxycinnamic acid, ferulic acid 4-glucoside, and their precursor, coumaric acid, were downregulated in stems, while the levels of isopulegone caffeate, ferulic acid 4-glucoside, and coumaric acid were increased in leaves under the N_0.1× condition. In particular, caffeic acid and its derivative isopulegone caffeate had accumulated in leaves under the K_0.1× and Mg_0.1× conditions compared with the control group. In the flavonoid biosynthetic pathway, in which flavonoids are biosynthesized from *p*-coumaroyl-CoA, the N_0.1× condition led to the accumulation of some flavonoids in leaves, including liquiritigenin, chrysin, scutellarein-7-*O*-glucuronide, and apigenin-7-*O*-glucuronide. However, some flavonoids, such as liquiritigenin, chrysin, scutellarein-7-*O*-glucuronide, and luteolin-7-*O*-glucoside, were downregulated under the K_0.1× condition and, also, the levels of chrysin, luteolin-7-*O*-glucoside, and apigenin-7-*O*-diglucuronide were decreased under the Mg_0.1× condition. In particular, the levels of tanshinones, derivatives of phenanthrenequinone, were downregulated under all mineral supply conditions in the leaves compared with the control group.

### 2.3. Comparison of Physicochemical Parameters and Metabolic Variations under Different Supplemental LED Lighting Conditions (Red, Blue, and Combination)

**Figure 4:**
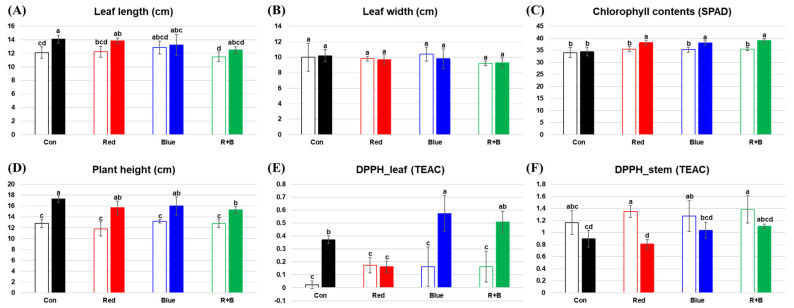
Leaf length (**A**), leaf width (**B**), chlorophyll content (**C**), plant height (**D**), and antioxidant activity (**E,F**) under different supplemental LED lighting conditions. Empty bars represent 10 days of supplemental LED lighting, and filled bars represent 21 days of LED lighting. Different letters indicate significant differences according to Duncan’s multiple-range test by analysis of variance (ANOVA, *p*-value < 0.05). TEAC: trolox equivalent antioxidant capacity; Con: control; R: red; B: blue.

We measured the physicochemical parameters of leaf length, leaf width, chlorophyll content, plant height, and antioxidant activity under different supplemental LED lighting conditions in *P. frutescens* (Figure 4). In the control group, plant height and antioxidant activity in leaves significantly increased from 10 to 21 days of the experimental time period, in which plants grew and their antioxidant activity increased. After 10 days under different LED lighting conditions, there were no significant differences in any of the parameters, i.e., leaf length, leaf width, chlorophyll content, plant height, and DPPH, in the leaves and stems. After a prolonged period (21 days) under different LED lighting conditions, DPPH in the leaves was lower in the red group but higher in the blue group and red–blue combination group compared with the control group, and other parameters were not dependent on the lighting condition.

**Figure 5:**
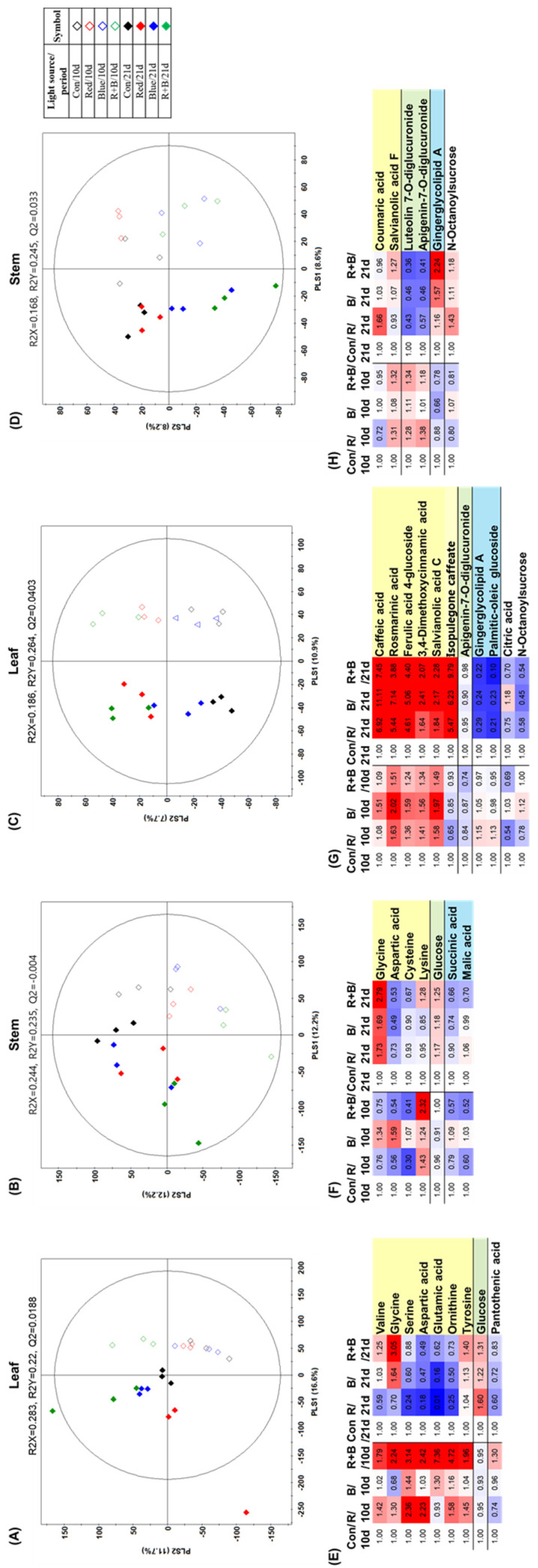
PLS-DA score plots derived from GC–TOF-MS datasets (**A**,**B**) and UHPLC–LTQ-MS/MS datasets (**C**,**D**) under different supplemental LED lighting conditions in *Perilla frutescens*. Significantly altered metabolites (VIP > 1.0 and *p*-value < 0.05) are represented by fold change from GC–TOF-MS datasets for leaves (**E**) and stems (**F**) and UHPLC–LTQ-MS/MS datasets for leaves (**G**) and stems (**H**). Con: control; R: red; B: blue; R+B: red–blue combination.

To identify the altered *P. frutescens* metabolites under different supplemental LED lighting conditions in leaves and stems, we also performed non-targeted metabolite profiling using GC–TOF-MS and UHPLC–LTQ-MS/MS with multivariate statistical analysis. The PLS-DA score plots from GC–TOF-MS and UHPLC–LTQ-MS/MS datasets (Figure 5A–D) exhibited distinct clustering for each group as well as for the different kinds of LED lighting and days of treatment.

A total of 29 metabolites, including 9 amino acids, 2 organic acids, 8 cinnamic acids, 2 flavonoids, 2 lipids, glucose, 3 unsorted metabolites, and 3 unidentified metabolites, were significantly altered under supplemental LED lighting. We have presented the differences in the abundance of metabolites for each supplemental LED lighting condition using heat map analysis (Figure 5E–H). In the case of supplemental LED lighting for 21 days, the levels of most amino acids in leaves were decreased by red LED lighting supplementation, while blue and red–blue combination LED lighting significantly increased some amino acids in leaves (Figure 5E). In stems, the levels of some organic acids (succinic acid and malic acid) and amino acids (aspartic acid and cysteine) were significantly downregulated, while glycine and lysine had accumulated under all supplemental LED lighting conditions compared with the control group (Figure 5F). For secondary metabolites derived from UHPLC–LTQ-IT-MS/MS data, the levels of most cinnamic acid derivatives, including rosmarinic acid, were increased in leaves, while apigenin-7-*O*-diglucuronide and some lipids were decreased under all supplemental LED lighting conditions (Figure 5G). In stems (Figure 5H), some flavonoids, including luteolin 7-*O*-diglucuronide and apigenin-7-*O*-diglucuronide, were decreased under all supplemental LED lighting conditions. The results of our metabolomics approach reveal that various mineral deficiency and supplemental LED lighting conditions might be linked to distinct metabolic features in the leaves and stems of *P. frutescens*, and further investigation is needed to explain the characteristic metabolites of each experimental condition at the molecular level.

## 3. Discussion

In this study, the N_0.1× condition significantly altered various metabolites and decreased the leaf length, leaf width, chlorophyll content, and antioxidant activity compared with other mineral-deficient conditions (Figure 1). According to previous reports, nitrogen deficiency led to significant reductions in leaf area, chlorophyll content, and the expression of photosynthesis-related genes, while resulting in induction of many genes related to the secondary metabolism of phenolics and the accumulation of flavonoids in plant tissues [15,16,17]. Nitrogen deficiency leads to reprogramming of the primary and secondary metabolism, and it was reported that the levels of amino acids and TCA cycle intermediates were found to be decreased using a metabolomics approach [18,19]. However, the overall underlying mechanism of nitrogen deficiency remains unclear. According to the metabolic pathway under the N_0.1× condition (Figure 4), the levels of most amino acids and organic acids derived from the TCA cycle were downregulated in *P. frutescens*, which was in line with previous observations [18,19]. Moreover, the shikimate and phenylpropanoid pathway were also analyzed in *P. frutescens* under nitrogen-deficient conditions. Most of the secondary metabolites had changed markedly in both leaves and stems under nitrogen-deficient conditions. Among them, the levels of most cinnamic acid derivatives derived from DOPA and coumaric acid were upregulated, and some flavonoids also increased under nitrogen-deficient conditions, except for salvianolic acid C, 3,4-dimethoxycinnamic acid, and apigenin-7-*O*-diglucuronide. Most flavonoids and phenolic compounds are potent inhibitors of oxidative damage due to their phenolic hydrogen, and the contents of these metabolites are increased in response to abiotic stresses. The initial step of phenylpropanoid metabolism is mediated by phenylalanine ammonia-lyase (PAL) and this enzyme can release ammonium ions from phenylalanine and structures of cinnamic acid could contribute to increases in different phenolic compounds under N-deficient conditions [15,16]. These results suggest that activation of secondary metabolism and downregulation of organic acids and amino acids under nitrogen deficiency is an integral part of plant adaptation to survive in a changed environment.

Under low potassium or magnesium supply conditions, there were no significantly alterations in phenotype compared with the control group, including in leaf length, leaf width, chlorophyll content, and plant height (Figure 1). However, the antioxidant activity in the stem was significantly decreased under low K and Mg conditions compared with the control group. As K deficiency does not immediately result in visible symptoms, the initial response to K-deficient conditions may be reduced aquaporin activity, which suppresses the root hydraulic conductance and water supply to the growing stem for diameter expansion [20]. In long-term periods of K deficiency, leaf tips are known to show brown scorching and curling, mainly in older leaves. This may represent a survival strategy adopted by plants, and this sign could be associated with the oxidative degradation of chlorophyll by reactive oxygen species (ROS) in plants under K-deficient conditions [21]. According to one report, plants kept under Mg-deficient conditions had shorter roots, smaller shoots, and necrotic spots on the leaves due to abnormal physiological processes and a decline in chlorophyll and carbon fixation [22]. Mg deficiency in plants causes degradation of chlorophyll and also decreased photosynthesis due to low CO_2_ assimilation [23]. In this study, there was little alteration in phenotype under K- and Mg-deficient conditions compared with control. Under K deficiency, in both leaves and stems (Figure 3), the levels of carbohydrates (maltose and glucose), most amino acids derived from pyruvate, and tyrosine were significantly increased, while most fatty acids, lipids, and their precursor (glycerol) were significantly downregulated, except for n-octanoylsucrose. The levels of rosmarinic acid derivatives also increased, while levels of most flavonoids derived from coumaric acid decreased under the K_0.1 condition. Armengaud et al. reported that metabolite changes under K deficiency generally resulted in increased carbohydrates and amino acids in *Arabidopsis* shoots through reduced activities of nitrogen reductase, glutamine synthetase, and glutamate synthase or through increased protein degradation [24], and our results are in accordance with these findings. For the low Mg supply, there were few significantly altered metabolites in the metabolic pathway (Figure 3). Nonetheless, the levels of rosmarinic acid derivatives, caffeic acid derivatives, and their precursors in addition to coumaric acid were significantly increased in leaves under the Mg_0.1× condition. Plant secondary metabolites play a major role in the adaptation to and defense against the environment in response to various abiotic stresses. Accumulation of secondary metabolites occurs in plants subjected to stresses, including various signal molecules [25].

Under different supplemental LED lighting conditions, most phenotypes in *P. frutescens*, including leaf length, leaf width, and plant height, had little change, while antioxidant activity in leaves and chlorophyll content increased under all supplemental LED lighting conditions compared with the control group (Figure 4). Many researchers have reported that red and blue LED lighting enhances plant biomass and improves economic characteristics by stimulating plant metabolism [26]. Some researchers have reported that increasing light intensity substantially increased total polyphenol, chlorophyll content, and rosmarinic acid in *P. frutescens* [27,28], and that artificial red and blue illumination induced the accumulation of rosmarinic acid and caffeic acid in red perilla [28]. In our study, the levels of amino acids decreased in leaves under supplemental red lighting but increased under supplemental red–blue combination lighting. The levels of most cinnamic acid derivatives increased in leaves, while some flavonoids and lipids decreased under all the different lighting conditions (Figure 5). Light insufficiency is a major abiotic stress limiting plant growth and crop yield in greenhouse production. It was recently demonstrated that these problems can be solved by growing medicinal and edible plants under controlled environments with supplemental LED lighting [29]. Our results did not show that supplemental LED lighting significantly improved the yields and the total volume of the green mass produced (data not shown). However, our results suggested that supplemental LED lighting could help in improving the plant quality in greenhouse cultivation, which could be important information for understanding the biosynthesis under different LED supplemental lighting conditions. Based on the current study, we suggest using a limited nutrient supply and supplemental light supply that includes blue color to produce perilla with enhanced levels of bioactive metabolites. Further study is needed to integrate other omics data, such as genomics and transcriptomics, which would allow an overall understanding of the mechanisms underlying *P. frutescens* plant responses to various environmental factors to be used in controlling growth conditions and enhance its productivity and quality.

## 4. Materials and Methods

### 4.1. Chemicals and Reagents

Methanol, ethanol, water, and acetonitrile were obtained from Fisher Scientific (Pittsburgh, PA, USA). Potassium persulfate, 2,2-diphenyl-1-picrylhydrazyl (DPPH), 6-hydroxy-2,5,7,8-tetramethylchromane-2-carboxylic acid (Trolox), formic acid, and standard compounds were obtained from Sigma Chemical Co. (St. Louis, MO, USA).

### 4.2. Plant Materials, Growth Conditions, Mineral Supply, and LED Treatment

Perilla (*Perilla frutescens* var. *japonica*, cv. Sangyeop; RDA, Jeonju, Korea) seedlings, which were 7-weeks old from germination, were transplanted into plastic pots (4.8 L) containing perlite and peat moss (1:1, *v*/*v*). They were then grown for either 25 or 36 days at 22 ± 6 °C in a greenhouse at the National Institute of Agricultural Sciences, RDA in Jeonju, Korea. They were illuminated by lamps from 18:00 to 21:00 in order to avoid flowering by extending the light period beyond natural sunlight. They were supplied daily with Hoagland solution consisting of 2.5 mM Ca(NO_3_)_2_, 2.5 mM KNO_3_, 1 mM MgSO_4_, 0.25 mM KH_2_PO_4_, 0.03 mM Fe-EDTA, 0.5 mM NH_4_NO_3_, 2 μM H_3_BO_3_, 0.2 μM MnCl_2_, 0.19 μM ZnSO_4_, 0.01 μM CuSO_4_, and 0.03 μM H_2_MoO_4_.

To test the effects of nutrient supply, plants at 15 days after transplantation were fed solutions with varying concentrations of either N, K, or Mg (either 0.1× or 0.75× that of Hoagland solution), which were produced by reducing the amount of the targeted nutrient in the solution and compensating for the reduced non-targeted nutrients—Ca(NO_3_)_2_, KNO_3_, and NH_4_NO_3_ were reduced and CaCl_2_ and KCl were added for reduced N supply; KNO_3_ and KH_2_PO_4_ were reduced and NH_4_NO_3_ and NaH_2_PO_4_ were added for reduced K supply; MgSO_4_ was reduced and K_2_SO_4_ was added for reduced Mg supply. After 10 days of low mineral supply, 6 leaves from the 3rd to the 5th node and a stem were each harvested from each plant. After 21 days of low mineral supply, 6 leaves from the 4th to the 6th node and a stem were harvested from each plant. For control plants, normal Hoagland solution was supplied, while other plants were supplied with reduced nutrient supply. Samples were harvested at 10:00 to avoid diurnal fluctuation of physiological responses (3 plants for each experimental condition).

To test the effects of LED treatment, perilla seedlings were grown under natural light until light treatment was initiated. From the 15th day after transplantation, the plants were placed under LED structures. The photosynthetic proton flux density (PPFD) of the natural light reaching the plants was lower than 500 μmol m^−2^s^−1^ as it was hindered by the LED structure. The LED light was switched on for 6 h/day (10:00–16:00), and it irradiated the plant canopy at a PPFD of 36 μmol m^−2^s^−1^. The LED lights radiated blue (455 nm, B), red (660 nm, R), or a combination of red and blue (660 and 455 nm, respectively; Com) light. The control plants were placed under the LED structure; however, the LED was not switched on. Light treatment continued for either 10 or 21 days. After 10 days of LED treatment, 6 leaves from the 3rd to the 5th node and a stem were harvested from each plant. After 21 days of LED treatment, 6 leaves from the 4th to the 6th node and a stem were harvested from each plant. Samples were harvested at 10:00 to avoid the diurnal changes in metabolites (3 plants for each experimental condition). Summarized information about the experimental conditions is listed in Table 1.

### 4.3. Measurement of Physiological Parameters and Antioxidant Activity

Plant height and the width and length of harvested leaves were measured at every harvest. Chlorophyll content from the leaves was measured using a chlorophyll meter (SPAD-502Plus, Konica Minolta Sensing, Osaka, Japan). The measured leaves were from the 3rd and 4th nodes from the bottom for the 10-day treatment, and from the 4th and 5th nodes from the bottom for the 21-day treatment.

The DPPH assay procedure was performed as described by Jung et al. [30] with some modifications. Twenty microliters of each sample was mixed with 180 μM of 0.2 mM DPPH ethanol solution in 96-well plates for 20 min at room temperature. Absorbance was measured at 515 nm using a microplate reader (Spectronic Genesys 6, Thermo Electron, Waltham, MA, USA).

### 4.4. Metabolite Extraction

The freeze-dried *P. frutescens* was pulverized using a mortar and pestle with liquid nitrogen. Each powdered sample (100 mg) was extracted with 70% aqueous ethanol (1 mL, *v*/*v*) followed by sonication for 10 min and shaking (30 S^−1^) for 10 min using an MM400 mixer mill (Retsch^®^, Haan, Germany). After extraction, the extract was centrifuged at 12,578× *g* for 10 min at 4 °C (Hettich Zentrifugen Universal 320, Tuttlingen, Germany), and supernatants were filtered using a 0.2-μm polytetrafluoroethylene filter. Samples were completely dried using a speed vacuum concentrator (Modulspin 31; BioTron, Inc., Seoul, Korea). The final concentration of all analytes was 10,000 ppm (10 mg/mL) using 70% aqueous ethanol.

### 4.5. Metabolomics Analysis

Gas chromatography–time of flight-mass spectrometry (GC–TOF-MS, LECO, St. Joseph, MI, USA) analysis was conducted as previously described by Jung et al. [31], and 1 μL of derivatized sample with 10 μL of internal standard (2-chlorophenylalanine) was injected in splitless mode. Methoximation and silylation were performed for the GC–TOF-MS analysis. A Rtx-5MS column (i.d., 30 m × 0.25 mm, 0.25 μm particle size; Restek Corp., Bellefonte, PA, USA) was used with helium as the carrier gas. Ultra-high-performance liquid chromatography–linear trap quadrupole-tandem mass spectrometry (UHPLC–LTQ-MS/MS, Thermo Fisher Scientific, Waltham, MA, USA) and ultra-performance liquid chromatography–quadrupole-time of flight-mass spectrometry (UPLC–Q-TOF-MS, Waters Corp., Milford, MA, USA) analyses were performed according to Suh et al. [32], and 5 μL of sample with internal standard (galangin) were injected into LC-MS. A Syncronis C18 column (100 mm × 2.1 mm, 1.7 μm, Thermo Fisher Scientific, Waltham, MA, USA) and Waters ACQUITY BEH C18 column (i.d., 100 mm × 2.1 mm, 1.7 μm particle size, Waters Corp., Milford, MA, USA) were used for chromatographic separation, respectively.

### 4.6. Data Processing and Multivariate Statistical Analysis

All analyses were conducted for 3 biological replicates. For metabolomics analysis, GC–TOF-MS and UHPLC–LTQ-MS/MS raw data were converted to NetCDF format (*.cdf) using LECO Chroma TOF™ software (version 4.44, LECO Corp., St. Joseph, MI, USA) and Xcalibur software (version 2.00, Thermo Fisher Scientific), respectively. After conversion, peak alignment was processed using MetAlign software (version 041012, Wageningen, Netherlands, http://www.metalign.nl), and the resulting data were exported to an Excel spreadsheet. Multivariate statistical analysis was performed using SIMCA-P+ 12.0 software (Umetrics; Umea, Sweden). The significantly altered metabolites under mineral deficiencies in *P. frutescens* were selected based on variable importance projection (VIP) values, and significance was tested by analysis of variance (ANOVA) and t-test using PASW Statistics 18 software (SPSS, Inc., Chicago, IL, USA). Selected metabolites were tentatively identified by comparing mass spectra, retention times, mass fragment patterns, UV absorbances, and elemental compositions derived from UHPLC–LTQ-MS/MS and UPLC–Q-TOF-MS analyses considering standard compounds, an in-house library, and published references. The heatmap was constructed by MeV software (http://www.tm4.org/).

## 5. Conclusions

In this study, we performed non-targeted metabolite profiling and measured physicochemical properties to explain the different metabolic mechanisms underlying the plant response to different mineral supply conditions and supplemental LED lighting conditions in *P. frutescens*. Both 10 or 21 days of treatment were too short to cause a significant change in plant phenotypic characteristics, except for the lowest N supply for 21 days, but sufficiently long enough to cause variation in metabolites in leaves and stems according to the different supply conditions. Among them, the lowest nitrogen supply caused large metabolite alterations in primary and secondary metabolism compared with the other mineral conditions; the lowest N supply increased some flavonoid contents by a factor of up to 5. The lowest K or Mg supply slightly increased the contents of rosmarinic acid and some flavonoids at 10 and 21 days. LED lighting of blue color or red–blue combination increased antioxidant activity compared with the control without LED supplementation at 21 days. LED lighting of red color, blue color, or red–blue combination increased rosmarinic acid by a factor of five, seven, and four, respectively. Our metabolomics approach showed the pattern of overall metabolic responses to various environmental factors depending on the different plant parts of *P. frutescens*. These results indicated that a metabolomics approach can provide insights into the metabolic responses to mineral deficiency and supplemental LED lighting conditions in plants, and reveal the key metabolic pathways underlying the plant responses to these environmental factors.

## Figures and Tables

**Table 1:** Information about the experimental conditions.

Parts	Experimental Conditions		Concentration or Light Intensity ^a^	Periods
Leaves and Stems	Mineral deficiency	Mineral_control	1.0×	10 days and 21 days
		Nitrogen	0.75×, 0.1×	
		Potassium	0.75×, 0.1×	
		Magnesium	0.75×, 0.1×	
	LED supply	LED control	-	
		Red_LED	660 nm, 36 μmol m^−2^s^−1^	
		Blue_LED	455 nm, 36 μmol m^−2^s^−1^	
		Red+Blue_LED	455 nm and 660 nm, 36 μmol m^−2^s^−1^	

^a^ Concentration of mineral is expressed as a fold change value compared with normal concentration, and the light intensity of LED lighting was measured in the perilla canopy. N: nitrogen; K: potassium; Mg: magnesium; LED: light-emitting diode.

## Supplementary Materials

Table S1: Significantly different primary metabolites of perilla grown under different mineral nutrient supply conditions and supplemental LED lighting conditions analyzed by GC–TOF-MS, Table S2: Significantly different secondary metabolites of perilla grown under different mineral nutrient supply conditions and supplemental LED lighting conditions analyzed by UHPLC–LTQ-MS/MS and UPLC–Q-TOF-MS.
