# Supplementary material for: Characterization of Metabolic Changes under Low Mineral Supply (N, K, or Mg) and Supplemental LED Lighting (Red, Blue, or Red–Blue Combination) in Perilla frutescens Using a Metabolomics Approach
Source: Molecules. 2020 Oct 14;25(20):4714. doi: 10.3390/molecules25204714 (PMC7587346; doi:10.3390/molecules25204714)
Supplement: Supplementary file 1 [file molecules-25-04714-s001.pdf]

molecules

Supplementary information

Characterization of Metabolic Changes under Low Mineral Supply (N, K, or Mg) and Supplemental LED Lighting (Red, Blue, or Red–Blue Combination) in *Perilla frutescens* Using a Metabolomics Approach

Dong Ho Suh [1,†], Yangmin X. Kim [2,†], Eun Sung Jung [3], Seulbi Lee [2], Jinyong Park [1], Choong Hwan Lee [1,4,*] and Jwakyung Sung [5,*]

[1] Department of Bioscience and Biotechnology, Konkuk University, Seoul 05029, Korea; sdh14031988@naver.com (D.H.S); park4jy@hanmail.net (J.P.)
[2] National Institute of Agricultural Sciences, Rural Development Administration, Wanju 55365, Korea; yangmink@korea.kr (Y.X.K.); seulvi23@korea.kr (S.L.)
[3] Department of Systems Biotechnology, Konkuk University, Seoul 05029, Korea; jes708@naver.com (E.S.J)
[4] Research Institute for Bioactive-Metabolome Network, Konkuk University, Seoul 05029, Korea
[5] Department of Crop Science, College of Agriculture, Life and Environment Sciences, Chungbuk National University, Cheongju 28644, Korea
* Correspondence: chlee123@konkuk.ac.kr (C.H.L.); jksung73@chungbuk.ac.kr (J.S.)
Tel.: +82-2-2049-6177 (C.H.L.); +82-43-261-2512 (J.S.)
† These authors contributed equally to this work.

Table S1. Significantly different primary metabolites of perilla grown under different mineral nutrient supply conditions and supplemental LED lighting conditions analyzed by GC–TOF-MS.

RT [a]	Metabolites	TMS [b]	Unique Mass	Mass Fragments
		**Amino Acids**		
6.76	Valine *,#	2TMS	144	73,100,144,218
7.31	Leucine *	2TMS	158	59,73,86,102,116,158,218,232,260
7.53	Isoleucine *	2TMS	158	73,100,158,218
7.58	Proline *	2TMS	142	73,100,142,216
7.66	Glycine *,#	3TMS	174	59,73,86,100,117,133,147,158,174,248,276
8.15	Serine #	3TMS	204	59,73,100,116,133,147,174,188,204,218,278
8.4	Threonine *	3TMS	117	57,73,86,101,117,147,203,219,291
9.54	Aspartic acid *,#	3TMS	232	73,100,147,188,202,200,000
9.59	5-Oxoproline *	2TMS	156	59,73,84,100,133,147,156,230,258
9.83	Cysteine *,#	3TMS	220	59,73,100,116,132,147,163,204,220,294
10.32	Glutamic acid #	3TMS	128	56,73,84,100,114,128,147,204,230,246,258,348
10.42	Phenylalanine *	2TMS	218	59,73,100,117,130,147,160,177,192,204,218,266
11.8	Ornithine *,#	4TMS	174	59,73,86,100,130,142,174
12.03	Lysine *,#	4TMS	174	59,73,86,100,156,174,200
12.64	Tyrosine #	4TMS	218	59,73,100,117,133,147,179,218,280
		**Carbohydrates**		
7.34	Glycerol	3TMS	103	59,73,89,103,117,133,147,205,218
12.47	Glucose *	5TMS	205	59,73,89,103,117,147,189,205,229,319
12.58	Glucose *,#	5TMS	205	59,73,89,103,129,147,160,205,319
12.71	Adonitol *	5TMS	103	59,73,89,103,117,129,147,189,217,319
13.69	Myo-inositol *	6TMS	217	59,73,81,103,129,147,191,217,265,305
16.95	Lactose *	8TMS	204	59,73,103,129,147,169,191,204,217,243,271,305,331,361
17.3	Maltose *	8TMS	204	59,73,103,129,147,169,191,204,217,243,271,305,331,361
		**Organic Acids**		
7.68	Succinic acid *,#	2TMS	129	55,73,86,117,129,147,218,247,262
7.88	Propanoic acid *	3TMS	189	59,73,103,117,133,147,175,189,205,292,307
7.97	Fumaric acid *	2TMS	245	73,147,217,245
9.27	Malic acid *,#	3TMS	233	55,73,101,117,133,147,189,217,233,265,307,335
9.96	Pentanedioic acid *	3TMS	129	73,129,147,231,247,300
		**Purines & Pyrimidines**		
7.96	Uracil	2TMS	241	73,99,113,126,147,169,185,241,255
12.19	Adenine *	2TMS	264	73,84,192,206,264,279
16.35	Inosine *	4TMS	217	59,73,103,129,147,169,193,217,245,281
		**Fatty Acids**		
13.21	Palmitic acid	1TMS	117	73,117,129,185,313
14.26	Linoleic acid	1TMS	117	67,103,117,129,147,200,000
14.4	Stearic acid *	1TMS	117	55,73,117,129,145,185,201,341
		**Etc.**		
12.95	Pantothenic acid *,#	3TMS	103	73,103,117,129,147,200,000,000

a Retention time.

b Trimethylsilyl.

* Significantly altered metabolites under different mineral deficient conditions (VIP >1.0 and p value < 0.05).

# Significantly altered metabolites under different supplemental LED lighting conditions (VIP >1.0 and *p* value < 0.05).

Table S2. Significantly different secondary metabolites of perilla grown under different mineral nutrient supply conditions and supplemental LED lighting conditions analyzed by UHPLC–LTQ-MS/MS and UPLC–Q-TOF-MS.

UHPLC-LTQ-MS/MS						UPLC-Q-TOF-MS					
**RT [a]**	**Mass**	**Tentative Metabolites**	**Adduct**	**Mass Fragments**	**λ max**	***m/z***	**Elemental Composition**	**mDa**	**PPM**	**DBE**	**i-FIT**
**Cinnamic Acids**											
0.98	163	Coumaric acid *,#	[M-H]$^-$	163/145/117/62	218	163.0382	C9H7O3	−1.3	8	6.5	0.1
4.99	179	Caffeic acid *,#	[M-H]$^-$	179/135	323/244	179.0348	C9H7O4	0.4	2.2	6.5	0.8
8.58	359	Rosmarinic acid *,#	[M-H]$^-$	359/341/223/197/179/161	326/292	359.0762	C18H15O8	−0.5	−1.4	11.5	0.3
9.38	355	Ferulic acid-4-glucoside *,#	[M-H]$^-$	355/337/311/219/193	328						
10.14	207	3,4-Dimethoxycinnamic acid *,#	[M-H]$^-$	207/179/161/135	328	207.0657	C11H11O4	−0.6	−2.9	6.5	0
10.47	491	Salvianolic acid C *,#	[M-H]$^-$	491/447/311/267	205/326	491.0986	C26H19O20	0.8	1.6	17.5	3.2
10.66	313	Salvianolic acid F *,#	[M-H]$^-$	313/269/203/161	326						
11.04	315	Isopulegone caffeate #	[M-H]$^-$	315/300/271/195/119	215/325						
**Flavonoids**											
6.61	637	Luteolin-7-*O*-diglucuronide *,#	[M-H]$^-$	637/351/285/193	366/330/269	637.103	C27H25O18	−1.1	−1.7	15.5	2.3
7.97	447	Luteolin-7-*O*-glucoside	[M-H]$^-$	447/403/285/181	366/319/283	447.0917	C21H19O11	−1	−2.2	12.5	0.7
7.26	621	Apigenin-7-*O*-diglucuronide *,#	[M-H]$^-$	621/487/351/269	365/326/285/269	621.1097	C27H25O17	0.5	0.8	15.5	3.5
8.33	445	Apigenin-7-*O*-glucuronide *	[M-H]$^-$	445/401/365/307/269/175	328	445.0778	C21H17O11	0.7	1.6	13.5	0.7
7.73	461	Scutellarein-7-*O*-glucuronide *	[M-H]$^-$	461/425/415/381/357/327/285/263/175	366/316/287	461.0708	C21H17O12	−1.2	−2.6	13.5	3
12.59	253	Chrysin *	[M-H]$^-$	253/225/209	220/291	253.0499	C15H9O4	−0.2	−0.8	11.5	0.4
12.7	255	Liquiritigenin *	[M-H]$^-$	213/187/151/145	366/325/300/292	255.0651	C15H11O4	−0.7	−2.7	10.5	1
**Lipids and Derivatives**											
10.37	327	oxo-Dihydroxy-octadecenoic acid *	[M-H]$^-$	327/309/291/229/221	323/297						
10.82	329	Trihydroxy-octadecenoic acid *	[M-H]$^-$	311/293/229/211/171	326	329.2312	C18H33O5	−1.6	−4.9	2.5	0.2
13.13	721	Gingerglycolipid A *,#	[M+FA-H]$^-$	721/675/415/397	221/326						
14.12	311	Tanshinone II-B *	[M-H]$^-$	311/293/283/275/171	222						
15.53	295	Tanshinone IIA *	[M-H]$^-$	295/277/251/195/171	223						
14.48	699	Palmitic-oleic glucoside *,#	[M-H]$^-$	699/653/397	222						
**Etc.**											
1.07	191	Citric acid *,#	[M-H]$^-$	191/173/129/111/85	247/273	191.0192	C6H7O7	−1.4	−7.3	3.5	0
6.82	239	Syringic acid acetate	[M-H]$^-$	239/221/195/177	366/328/280						
8.57	719	Sagerinic acid *	[M-H]$^-$	719/556/522/359/285	326	719.1641	C36H31O16	2.9	4	21.5	3.5

8.83	467	N-Octanoylsucrose *,#	$[M-H]^-$	467/421/286	323/291
**N.I**					
7.63	449	N.I 1 *		449/403/269	285/328
10.46	527	N.I 2 *		527/491	326
11.45	667	N.I 3 *		667/505/311	302/326
12.11	625	N.I 4 *,#		625/463/311	292/326
12.51	325	N.I 5 *		325/307/263	219/323
13.81	723	N.I 6 *,#		723/677/397	222/328
14.57	559	N.I 7 *		559/513/277	225
15.53	561	N.I 8 *		561/515/279	223
15.57	529	N.I 9 *,#		529/511/279	223

a Retention time.

* Significantly altered metabolites under different mineral deficient conditions (VIP >1.0 and p value < 0.05).

# Significantly altered metabolites under different supplemental LED lighting conditions (VIP >1.0 and p value < 0.05).
